# A Case of Poisoning by Arsenic

**Published:** 1823-02

**Authors:** J. W. Edwards

**Affiliations:** Surgeon.


### Art. VII.—*A Case of Poisoning by Arsenic.* By J. W. Edwards, Esq. Surgeon.

E. T., of a firm and robust habit, ætat. 35, and advanced seven months in her pregnancy, was prevailed on, by the father of her infant, to take what he had procured for her, which (from the best information that could be collected) consisted of an ounce of white arsenic.* This she mixed in half a pint of hot water, and stirred the mixture well with a spoon; she then drank about the half of it, saying that she could not drink the whole, as her "stomach went against it." In about eight minutes after the poison had been swallowed, it began to affect her with excessive sickness, pains, and other alarming symptoms. Having been sent for, about eight o'clock in the morning, August 31st, I found her much exhausted from violent retching and vomiting. She complained of very great cold in the extremities, unquenchable thirst, spasmodic pains in the bowels, especially towards the epigastric region; the mouth was much parched; the eyes bloated, and the face evidently swollen; and she was also very restless, showing great anxiety and distress of mind. The pulse at that time 120.

I lost no time in administering the *carbonate of magnesia* as an antidote to the poison, and followed the same plan as recommended by Mr. Hume, of Long-Acre, who, I believe, first mentioned it as a proper remedy. I likewise adopted the same prescription, which I find in the London Medical and Physical Journal for November, 1821,—namely,

℞. Magnesiæ Carbonatis, ℥j.

Vini Opii, ʒjss.

Sacchari albi, ℥ss.

Aquæ distillatæ, ℔j. Misce fiat mistura.

Of this I ordered a wine-glassful to be taken every fifteen minutes, shaking the bottle; and that this should be accompanied with a free use of mucilaginous drinks, such as gruel, barley-water, broths, &c. taken in small but frequent draughts. I also directed the proper means to keep the extremities warm.

* My own experiments to prove the identity of this poison were fully confirmed by Mr. Hume, to whom I sent a very small portion for analysis.

At noon, the same day, I again saw the patient. The vomiting was less frequent, and not so violent; the skin was very hot; she complained of intense thirst, a burning pain in the stomach, and great soreness on pressure; pains of the head and sides, and great restlessness. Pulse 136, and strong.—Venesectio e brachio ad uncias viginti. Repetatur mistura magnesiæ carbonatis. As there was an evident remission of the more urgent symptoms, I ordered the mixture to be taken every half hour only, but still in the same dose.

In the evening, about seven o'clock, I found her more calm. She complained of great soreness of the throat and mouth; the vomiting and retching were less urgent; she felt pain over the region of the stomach, and towards the right side. Pulse 106. —Habeat Olei Ricini, ℥j. statim. Applicentur hirudines xii. lateri dolenti. Perstat in usu misturæ.

*September* 1*st.*—I saw her at eight o'clock A.M. She had slept about three hours in the course of the night; the vomiting had abated; the burning sensation of the throat and stomach had materially diminished, and the bowels had been scantily purged. Pulse 104.

℞. Olei Ricini, ℥jss.
Mucilaginis Acaciæ, ℥vj. Misce ut fiat mistura cujus sumat cochlearia duo majora tertiis horis donec alvus satis soluta fuerit; postea continuetur mistura, omittando vinum opii.

*September* 2*d.*—She complained much of soreness in the throat and stomach; the bowels are open; the skin is moist; she had been faint several times during the night; her pulse 98, and small.—Ordered the magnesia mixture, as last prescribed, to be continued every two hours; and small quantities of weak broth to be taken frequently.

*September* 3*d.*—The patient continues to improve, and advance, although slowly, towards convalescence. I prescribed a milk-diet; the mixture to be taken three or four times a-day; and that the bowels should be kept moderately open.

The distance between this town and Kingsbridge Poor-house, in which the patient is an inmate, precludes me from rendering this case so perfect and circumstantial as might be required by your readers. I have this day (November 6th,) seen the woman, and find that the chief symptoms now remaining are, from her own report, indistinct vision; great weakness in the stomach; a numbness extending from the feet to the knees; that her hands are similarly affected, and are always cold; and that she is so faint that she thinks she will never be quite well again. Notwithstanding these sequelæ, and so much disturbance to which this patient's constitution had already been exposed, abortion has not taken place, nor is there at present any reason to apprehend such an event: on the contrary, she describes her feelings to indicate that "the child still continues to be very strong."

Although this woman is married, yet the infant must be illegitimate, her husband having, as a convict, been some time ago transported. The reputed father, in the present case, had pretended that he himself was affected with the venereal disease, and persuaded the mother to take the powder, that she might be secured from the infection. His having absconded, and from other circumstances, there is no doubt, however, that his intentions were of a more serious description.

*Chillington, near Kingsbridge, Devon; Nov.* 6, 1822.

